# From the gut to the lungs: The role of gut microbiota in chronic obstructive pulmonary disease and related research progress

**DOI:** 10.15698/mic2026.04.873

**Published:** 2026-04-14

**Authors:** Simin Yang, Shuting Zeng, Yongan Deng, Xiaodong Duan, Chengkai Chen, Luyun Sun, Yongkang Qiao, Zunpeng Shu

**Affiliations:** 1 School of Chinese Materia Medica, Guangdong Pharmaceutical University, Guangzhou, 510006, China; 2 Key Laboratory of Cell Proliferation and Regulation Biology, Ministry of Education, Department of Biology, Faculty of Arts and Sciences, Beijing Normal University, Zhuhai, 519087, China

**Keywords:** COPD, gut microbiota, gut-lung axis, gut microbiota metabolites, immune regulation

## Abstract

Chronic Obstructive Pulmonary Disease (COPD) is a progressive respiratory disease with high morbidity and mortality. Existing treatment methods are difficult to effectively curb disease progression, highlighting the urgency to explore new pathogenesis mechanisms and therapeutic targets. With the development of microbiomics, the proposal of the “gut-lung axis” concept has provided a brand-new perspective for understanding the pathological mechanisms of COPD, revealing that the gut and lungs maintain a close connection through pathways such as immune regulation and metabolic interaction. This article systematically elaborates on the association between gut microbiota and COPD: First, it deeply analyzes the pathological interaction between the gut and lungs from the perspective of the gut-lung axis. On this basis, it examines the characteristic changes in gut microbiota and their metabolites in COPD patients, explores the key influencing factors driving such microbiota dysbiosis, and further systematically explains the core mechanisms by which gut microbiota contribute to the occurrence and progression of COPD. Finally, it focuses on strategies for the prevention and treatment of COPD based on gut microbiota regulation, and prospects their clinical application potential. The purpose of this article is to provide new ideas and directions for the basic research and clinical practice of COPD by comprehensively sorting out the association between gut microbiota and COPD, thereby helping to improve the current status of COPD prevention and treatment.

## INTRODUCTION

As the third leading cause of death worldwide 1, Chronic Obstructive Pulmonary Disease (COPD) is a progressive respiratory disorder characterized by persistent airflow limitation 2 and has emerged as a major global public health challenge 3. According to statistics from the World Health Organization (WHO), COPD has remained consistently high in morbidity and mortality 4. It not only severely impairs patients’ lung function and quality of life but also is accompanied with a variety of systemic comorbidities (*e.g.*, pulmonary hypertension 5, osteoporosis 6, metabolic syndrome 7), imposing a heavy medical burden on families and society 8. Currently, the clinical management of COPD mainly relies on symptomatic interventions such as bronchodilators and anti-inflammatory drugs 9. However, existing strategies are unable to effectively halt disease progression or reverse lung function impairment, making in-depth exploration of its pathogenesis and the search for novel prevention and treatment targets an urgent priority in the medical field 10.

In recent years, with the vigorous development of microbiomics research, the association between the human microbiota and diseases has gradually become a research hotspot. As the largest microecosystem in the human body, the gut has been proven to have a intimate crosstalk between the balance and dysbiosis of its microbial structure and the development of various chronic diseases 11. Moreover, the proposal of the “gut-lung axis” concept has further broken the traditional understanding that respiratory diseases are confined to local pulmonary research, and revealed the close link between the gut and the lungs via multiple pathways such as immune regulation 12, 13, metabolic crosstalk 14, 15, and neuroendocrinology 16–18. This groundbreaking perspective provides a novel dimension for elucidating the complex pathological mechanisms of COPD; mounting evidence suggests that gut microbiota dysbiosis may be involved in the onset, progression, and acute exacerbation of COPD by modulating systemic inflammatory responses, immune dysregulation, and metabolic abnormalities 19.

Based on the above evidence, this review systematically elaborate on the association between gut microbiota and COPD. Starting from the physiological and pathological crosstalk of the gut-lung axis, it will analyze the characteristic alterations in gut microbiota and their metabolites in patients with COPD, explore the key factors driving microbial dysbiosis and their mechanisms of action in the disease process, and further discusses and prospects the preventive and therapeutic strategies of COPD based on the gut microbiota modulation as well as their application potential. The primary aim of this review is to provide novel ideas and directions for the basic research and clinical practice of COPD.

## THE ASSOCIATION BETWEEN THE GUT AND LUNGS FROM THE PERSPECTIVE OF THE GUT-LUNG AXIS AND THE PATHOLOGICAL INTERACTIONS IN COPD

The epithelial structures of the gut and lungs exhibit striking structural similarities: the gut is composed of columnar epithelial cells with apical surface microvilli, while the lungs are lined with ciliated columnar epithelial cells. Both form physical barriers via tight junctions to block pathogen invasion and simultaneously act as immune sentinels 20. Additionally, they are part of the common mucosal immune system: both secrete mucus through multiple cell types to trap pathogens and rely on immunoglobulins to defend against bacterial and viral attacks 21. Furthermore, the gut microbiota and lung microbiota do not exist in isolation, and their dynamic ecological changes exhibit pronounced interorgan synchrony: the gut microbiota mediates remote pulmonary regulation through systemic pathways, whereas the airway microbiota itself undergoes intrinsic ecological shifts. This coordinated interaction between the two processes provides core mechanistic support for the physiological homeostasis and pathological dysfunction of the gut-lung axis 22.

**Figure 1:**
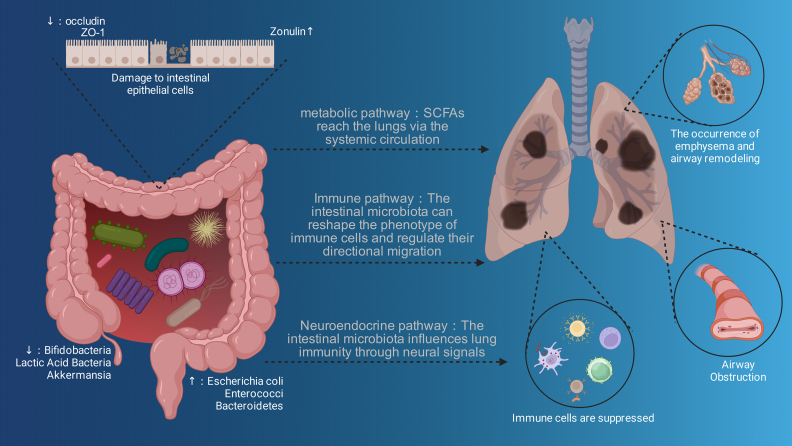
The Multi-Pathway Mechanism of Gut Microbiota Regulating COPD Mediated by the Gut-Lung Axis. Gut microbiota mediates the regulation of COPD via multiple pathways of the gut-lung axis, while intestinal epithelial damage and microbiota alterations influence COPD-related lung conditions such as emphysema and airway obstruction.

The gut microbiota constitutes the core mechanism of the gut-lung axis through the remote regulation by metabolites and the cross-organ interaction with immune cells. At the metabolic level, animal model studies have shown that gut microbiota-derived metabolites, such as short-chain fatty acids (SCFAs; acetate, butyrate, propionate), secondary bile acids, and tryptophan metabolites, can act on the lungs via the systemic circulation 23, 24. Among these, SCFAs can reduce the release of IL-6 and TNF- α by inhibiting the NF- κ B pathway 25, 26, while butyrate treatment can more significantly alleviate lung inflammation and excessive mucus production in ovalbumin (OVA)-challenged mice 27. At the immunological level, the gut microbiota can regulate pulmonary immune function through the gut-modulated pulmonary immune network. For instance, *Tritrichomonas musculis*, a commensal protozoan in the gut, can drive the migration of group 2 innate lymphoid cells (ILC2s) to the lungs in mice 28. This phenomenon plays a pivotal role in modulating the pulmonary immune microenvironment and influencing the outcomes of airway lesions, and highlights the critical position of gut microbiota members in mediating the host’s response to airway challenges through targetable molecular pathways. Notably, terms such as “neuroendocrinology” and “gut-lung-brain axis” have garnered increasing attention in this field in recent years 29, 30. For instance, vagus nerve stimulation (VNS) exerts a protective effect on the intestinal barrier and prevents acute lung injury (ALI) following traumatic hemorrhagic shock by stimulating the enteric nervous system 31. As one of the twelve pairs of cranial nerves, the vagus nerve connects the brain to all vital organs of the organism via multiple organs, including the esophagus, heart and lungs 32. Increased excitability of the vagus nerve improves intestinal barrier function and downregulates the pulmonary inflammatory cascade, thereby alleviating lung tissue injury 33. In addition, the gut microbiota can regulate the Hypothalamic-Pituitary-Adrenal Axis (HPA axis). Dysregulation of the HPA axis may elevate the levels of cortisol and pro-inflammatory molecules, thereby impairing the integrity of the intestinal barrier, leading to bacterial translocation into the bloodstream and causing subsequent chronic inflammation in the central nervous system, which in turn impairs cognitive function 34, 35. Meanwhile, pulmonary pathophysiological abnormalities and impaired mucus clearance mechanisms trigger dysbiosis, which induces the translocation of bacteria and endotoxins via the lung-gut axis, further exacerbating central nervous system inflammation and cognitive impairment 36. The gut microbiota plays a central regulatory role in the progression of lung injury to cognitive impairment. These pathological processes all unfold around the gut-lung-brain axis as the core regulatory network and rely on its associated neuroendocrine pathways to achieve cross-organ regulation. Clearly, these interactions highlight a new dimension of bidirectional communication between the gut microbiota and other organs such as the lungs, and all play pivotal roles in maintaining overall health (Figure 1).

The close pathological crosstalk mediated by the gut-lung axis can bidirectionally exacerbate the progression of both COPD and intestinal diseases, which has become a crucial factor contributing to the increased comorbidity rate of these two types of disorders. As is well known, cigarette smoke (CS) exposure is a classic and mainstream method for establishing COPD animal models 37, with whose advantage lying in the high simulation of the primary pathogenic factors and pathophysiological characteristics of human COPD 38. Existing studies have indicated that long-term smoking can alter the microcirculation system, significantly reduce blood flow in the gastrointestinal mucosa 39 and inhibit gastrointestinal angiogenesis 40, even promoting the abnormal proliferation of pro-inflammatory bacteria 41. Meanwhile, while patients with COPD often use antibiotics against pathogenic bacteria in the lungs, this practice further exacerbates microbiota dysbiosis 42, 43. This imbalance triggers intestinal barrier damage, which is manifested by elevated plasma Zonulin levels (a marker of intestinal leakage) in COPD patients—with levels significantly higher in patients with moderate-to-severe COPD than in those with mild disease. This allows harmful substances to enter the bloodstream and induce systemic inflammation 44. Furthermore, the systemic inflammatory state of COPD interferes with intestinal peristalsis, absorption, and immune function through factors such as TNF- α and IL-6, exacerbating gastrointestinal symptoms including abdominal pain and diarrhea 45. Conversely, intestinal dysfunction can also exacerbate the progression of COPD: impairment of the intestinal barrier leads to the translocation of bacteria (*e.g.*, *Pseudomonas aeruginosa*) 46 and endotoxins 47 to the lungs, increasing the risk of pulmonary infection. Mendelian randomization studies have confirmed the bidirectional causal relationship between them: gastroesophageal reflux disease (GERD) can increase the risk of developing COPD, and COPD can also raise the incidence of irritable bowel syndrome (IBS) and constipation 48. In summary, the bidirectional association between COPD and intestinal diseases mediated by the gut-lung axis is essentially a cross-organ closed-loop cascade reaction driven by gut dysbiosis-induced disorders in the “metabolism-immunity-neuroendocrine” network. Specifically, the shift in gut microbiota composition leads to a deficiency of beneficial metabolites and accumulation of pro-inflammatory substances, which impairs the intestinal and pulmonary barriers and triggers systemic inflammation. In turn, the inflammation microenvironment associated with COPD and the abuse of antibiotics further exacerbate gut dysbiosis, thereby forming a pathological cycle. This mechanism reveals the core cause of the high comorbidity rate between the two diseases, identifies gut microbiota as a key node linking local lesions to systemic responses, provides theoretical support for developing novel microbiota-targeted therapies for COPD, and also highlights the urgency of conducting large-sample studies to clarify the key targets of microbiota-host interactions.

## CHARACTERISTIC CHANGES IN GUT MICROBIOTA AND METABOLITES IN PATIENTS WITH COPD

Dysregulation of the gut microbiota and its metabolites is regarded as a key feature in the pathophysiological process of COPD. A large body of clinical studies has confirmed that patients with COPD exhibit significant structural dysbiosis of the gut microbiota and abnormalities in metabolites. These changes not only reflect the disease state but also are likely to be involved in the occurrence and progression of the disease.

### Changes in the overall structure of the gut microbiota

The overall gut microbiota of COPD patients exhibits characteristic changes, which serve as an important marker of microbiota structural dysbiosis. Multiple cross-sectional studies have compared gut microbiota diversity between COPD patients and healthy controls, and the results show that the α -diversity of gut microbiota in COPD patients is significantly reduced—with the most consistent decreases observed in the Chao1 index (reflecting microbial richness) and the Shannon index (reflecting both microbial richness and evenness) 49, 50. A clinical study comparing patients with stable COPD and healthy subjects found that the Shannon index in the COPD group was lower than that in the control group, and this reduction was positively correlated with the percentage of predicted forced expiratory volume in 1 second (FEV ${}_{1}$ % pred) of lung function 51. In terms of β -diversity, results from Principal Coordinate Analysis (PCoA) showed that the gut microbiota structures of COPD patients and healthy individuals could be clearly divided into two clusters, indicating a significant difference in the overall composition of the microbiota between the two groups 52. Notably, age, smoking status, dietary patterns, and medication use are well-recognized confounding factors that also modulate these gut microbiota diversity indices, and thus their potential impacts need to be taken into account when interpreting clinical findings related to COPD and the gut microbiota 53.

At the specific genus level, the gut microbiota composition of COPD patients shows an imbalanced trend characterized by the enrichment of pro-inflammatory genera and the reduction of anti-inflammatory genera. This characteristic change has been verified in studies involving different ethnicities and regions. Regarding pro-inflammatory genera, the abundance of *Escherichia* in the gut of COPD patients is higher than that in the healthy control group 54, and the enrichment of this genus is positively correlated with serum IL-6 levels 55. Although the genus *Enterococcus* is not a common pathogen of pulmonary infections in patients with COPD, case studies have indicated that advanced-stage patients need to be alert to the pathogenic potential of its drug-resistant strains (*e.g.*, vancomycin-resistant *Enterococcus*, VRE), which is associated with airway structural damage and gut-lung axis dysfunction in these patients 56. As one of the dominant bacterial genera in the human gut, *Bacteroides* exhibits a significant alteration in its abundance in patients with COPD 57, and this change may further affect the host’s inflammatory status by disrupting the homeostasis of bile acid metabolism 58.

In contrast, genera with anti-inflammatory and metabolic regulatory functions show a characteristic reduction in the gut of COPD patients. A decrease in the abundance of *Bifidobacterium* is one of the most common changes; a meta-analysis shows that its abundance in the gut of COPD patients is lower than that in healthy individuals, and this reduction is more pronounced during acute exacerbation 59. *Lactobacillus rhamnosus*, a probiotic, can alleviate pulmonary inflammation and improve lung tissue remodeling in cigarette smoke-induced COPD mice, while regulating the balance between pro- and anti-inflammatory cytokines in bronchial epithelial cells 60. The genus *Akkermansia* exhibits decreased abundance in the gut of patients with smoking-related COPD, accompanied by altered gut microbiota metabolic phenotypes and abnormal retinol metabolism (changes in retinol and retinal levels), and it can ameliorate lung injury in cigarette smoke-induced COPD mice by regulating inflammation and autophagy 61. As a major producer of SCFAs, the abundance of the genus *Faecalibacterium* is significantly positively correlated with the level of butyrate in feces 62; this genus exhibits reduced abundance in patients with COPD, and its abundance further decreases with the progression of disease severity 63.

It should be noted that since most of the above studies are cross-sectional, the patients were already in a diseased state at the time of sample collection. Therefore, in terms of causality consideration in observational studies, it is difficult to determine whether the composition of the microbiome is a cause or a consequence of respiratory diseases. In addition, genus-level microbial changes may mask potential strain-level functional differences, and changes in the relative abundance of a specific genus do not always directly indicate a causal relationship with the pathogenesis of COPD. Distinct strains within a single genus can display divergent biological properties, and abundance shifts may also represent a secondary consequence of the host’s pathological state rather than a primary driving factor 64–66. In summary, disease-related host alterations and changes in the gut microbiota may interact and act simultaneously. Nevertheless, it is necessary to further explore the impact of the gut microbiota on the severity and progression of COPD in the future, which is expected to provide additional clinical implications.

### Abnormal changes in characteristic metabolites

Metabolites of the gut microbiota serve as a crucial bridge connecting the microbiota to the host’s physiological functions. Imbalance of the gut microbiota in patients with COPD directly leads to significant changes in metabolites, among which the alterations in metabolites related to immune regulation, inflammatory responses, and oxidative stress are the most critical.

SCFAs, the main products of dietary fiber fermentation by the gut microbiota 67, show a significant decreasing trend in patients with COPD, and this is one of the core features of abnormal microbiota metabolism. The reduction in SCFAs may affect the progression of COPD through multiple mechanisms: on the one hand, butyrate serves as the primary energy source for intestinal epithelial cells, and its deficiency can impair the integrity of the intestinal barrier 68. Studies have shown that the fecal butyrate level in COPD patients is positively correlated with the expression of occludin—a tight junction protein in the intestinal mucosa 69. On the other hand, SCFAs can inhibit the release of pro-inflammatory factors such as IL-1 β and TNF- α from macrophages by activating G protein-coupled receptors 70. In addition, treatment with SCFA reduces the secretion of LPS-induced IL-1 β and increases the secretion of IL-10 in human peripheral blood mononuclear cells (PBMCs), and this effect is mediated via the TLR4 signaling pathway 71.

Trimethylamine N-oxide (TMAO), a characteristic metabolite mediated by the gut microbiota, has a metabolic process that relies on the synergistic interaction between the gut microbiota and the host liver 72. The gut microbiota metabolizes dietary precursor substances such as choline and L-carnitine to produce trimethylamine (TMA); TMA is absorbed through the intestinal mucosa into the liver and oxidized to form TMAO under the catalysis of flavin-containing monooxygenase 3 (FMO3) 73. Clinical studies have indicated that circulating TMAO levels are significantly higher in patients with COPD than in healthy individuals 74. Similarly, clinical analyses have shown that elevated plasma TMAO levels are associated with long-term fatal outcomes in patients with community-acquired pneumonia (CAP) 75. Further studies have revealed that 3,3-dimethyl-1-butanol (DMB) can reduce TMAO levels and ameliorate pulmonary vascular remodeling as well as pulmonary artery smooth muscle cell (PASMC) proliferation in animal models of pulmonary hypertension (PH). The underlying mechanism is that TMAO promotes PASMC proliferation and migration by upregulating the secretion of inflammatory factors from macrophages, while DMB can inhibit this macrophage-mediated TMAO pathway that drives PH progression 76. In summary, the effects of TMAO on the vascular endothelial barrier may explain the mechanism by which TMAO mediates COPD, as impairment of the vascular endothelial barrier leads to the migration of macrophages, neutrophils, and protein-rich fluid into the alveoli 77–79. However, most existing studies have focused on the correlation analysis between TMAO levels and COPD, and there is a lack of direct mechanistic research to confirm the causal relationship between TMAO and the pathogenesis of COPD. In particular, in COPD patients, the specific gut microbiota strains involved in TMAO synthesis, as well as the impact of FMO3 gene polymorphisms on TMAO metabolism and the risk of COPD onset, still need to be further verified by large-sample clinical studies and animal experiments.

In addition, the tryptophan metabolic pathway of the gut microbiota in COPD patients also exhibits significant abnormalities 80. Tryptophan is metabolized by the gut microbiota to produce anti-inflammatory metabolites such as indole and indoleacetic acid 81, and the levels of these metabolites are significantly altered in patients with COPD 82, 83. Meanwhile, the metabolites of the host’s own kynurenine pathway (such as kynurenine and quinolinic acid) are elevated in the serum of COPD patients 84–86. This metabolic shift may be involved in the regulation of pulmonary inflammation by affecting the function of regulatory T cells 87 and the activation of the aryl hydrocarbon receptor (AhR) 88. Notably, GERD represents one of the most prevalent extrapulmonary manifestations in patients with COPD, with a tight pathological correlation established between them 89. The underlying mechanisms by which GERD exacerbates the pathophysiology of COPD have not yet been fully elucidated, but it may involve the translocation of gastric metabolites and/or microorganisms to the lower respiratory tract through the reflux-microaspiration process 90. As core gastrointestinal metabolites, bile acids (BAs) have been detected in the bronchoalveolar lavage fluid (BAL) of COPD patients in a clinical study 91. This finding suggests that BAs may be closely associated with COPD and, by virtue of their diverse biological properties, participate in the regulation of COPD-related pathophysiological processes, thus providing pivotal evidence for unraveling the functional link between gastrointestinal disorders and COPD. It should be recognized that research evidence pertaining to gut microbial metabolic dysregulation in COPD is still accumulating, and subsequent studies and investigations will further elucidate the precise mechanistic links between such metabolic abnormalities and the pathogenesis and progression of COPD.

## KEY INFLUENCING FACTORS DRIVING COPD-ASSOCIATED GUT MICROBIOTA DYSBIOSIS

**Figure 2:**
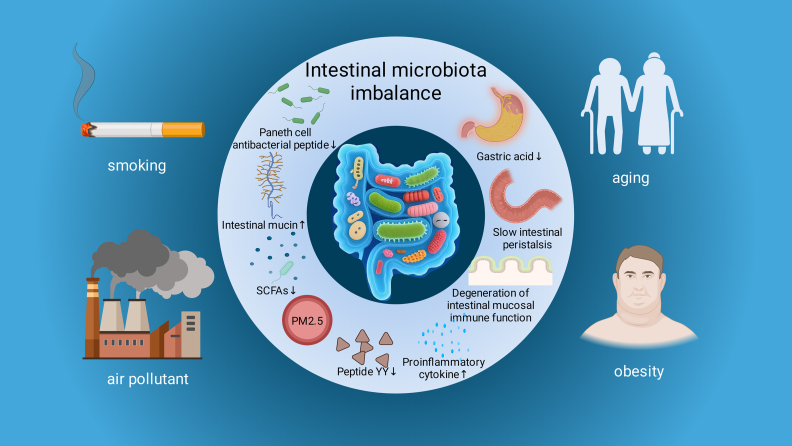
Driving Factors of COPD-Related Intestinal Dysbiosis. Schematic diagram illustrating factors (smoking, air pollutants, aging, obesity) driving COPD-related intestinal microbiota imbalance, along with associated alterations in intestinal physiology and metabolites.

Gut microbiota dysbiosis in patients with COPD is co-driven by exogenous environmental factors and endogenous host factors. By altering the intestinal microenvironment, immune status, and metabolic state, these factors disrupt microbial homeostasis and accelerate disease progression (Figure 2).

### Smoking

Smoking is a core factor inducing both COPD and gut microbiota dysbiosis 92, 93. Numerous studies have demonstrated that cigarette smoking exerts diverse and adverse effects on the initiation and progression of COPD 94. Smoking represents a major risk factor for COPD, with a clear dose-response relationship 95. Mechanistically, smoking impairs airway epithelial function, enhances oxidative stress by disrupting the antioxidant defense system and suppressing the activity of pulmonary antioxidant enzymes, and activates pro-inflammatory signaling pathways to promote the release of pro-inflammatory cytokines, thereby exacerbating persistent chronic airway inflammation 96. Smoking also accelerates small airway remodeling and emphysema formation, resulting in a progressive decline in key pulmonary function parameters such as the FEV ${}_{1}$ , and compromises pulmonary ventilation and gas exchange 97. Although smoking-related pulmonary impairment can be partially reversed upon smoking cessation, the irreversible tissue damage induced by long-term heavy smoking is not recoverable 98, 99.

Animal experiments have shown that exposure to CS increases the abundance of *Clostridium* in the intestines of mice, while reducing the levels of beneficial bacteria such as *Lactobacillus* and *Ruminococcus* 100, 101. Human studies have confirmed that smoking leads to gut microbiota dysbiosis 102. After smoking cessation, the abundances of *Firmicutes* and *Actinobacteria* increase, while those of *Bacteroidetes* and *Proteobacteria* decrease, and the gut microbiota structure gradually recovers 103. Smoking also reduces the content of SCFAs 104. However, *Firmicutes* can restore SCFA levels by metabolizing plant polysaccharides, thereby alleviating intestinal damage 105. In addition, CS can further exacerbate gut microbiota dysbiosis by altering the human mucosal environment. For instance, CS not only induces increased secretion of ileal mucins Muc2 and Muc3 and enhanced expression of Muc4 106, but also causes specific changes in Paneth cell granules, impairing their ability to produce antimicrobial peptides and exert bactericidal effects 107. In addition, CS disrupts tight junction proteins by activating the NF- κ B pathway, increases intestinal permeability, and triggers intestinal villus atrophy and bacterial translocation 107, 108. In summary, cigarette smoking serves as a core common factor contributing to both COPD and gut microbiota dysbiosis. It promotes the initiation and progression of COPD and causes irreversible lung injury through multiple pathways, and also induces gut microbiota dysbiosis by disturbing microbial structure, reducing SCFA levels, and impairing the intestinal mucosal barrier. Its dual effects make it difficult to clarify the causal relationship between COPD and gut microbiota. Future studies are warranted to further elucidate the regulatory mechanism of the gut-lung axis mediated by smoking, which may provide new strategies for the prevention and treatment of COPD via smoking cessation combined with gut microbiota intervention.

### Air pollutants

Air pollutants significantly increase the risk of acute exacerbation of COPD and disrupt the gut microbiota 109, 110. Li *et al*. identified six bacterial strains that are closely associated with air pollutants and blood biochemical indices, and these strains are linked to the clinical status of patients with acute exacerbation of chronic obstructive pulmonary disease (AECOPD). Among these findings, particulate matter with an aerodynamic diameter of ≤ 2.5 μ m (PM ${}_{\mathrm{2.5}}$ ) was positively correlated with *Clostridium sp*. CAG127 (a strain belonging to *Firmicutes*) and negatively correlated with the number of monocytes (Mono) 111. A study demonstrated that particulate matter (PM) enhanced the diversity of intestinal microbiota in the small intestine, colon, and feces, altered the composition of the gastrointestinal microbiota, with these changes exacerbating progressively from the proximal to the distal gastrointestinal tract 112. Among these changes, the abundance of *Clostridium* was significantly increased in mice exposed to air pollutants. In addition, the phylum Firmicutes was markedly reduced, which was consistent with the change trend observed in patients with inflammatory bowel disease (IBD) 113. An investigation focused on school settings and children exposed to environmental pollutants to explore the tripartite associations among heavy metal contamination, environmental microbiota, and the gut microbiome of children. Heavy metal contamination was identified in school environments, which significantly altered the community structure of environmental microbiota; notably, lead (Pb) and cadmium (Cd) exerted the most prominent effects on environmental microbial assemblages. Further findings confirmed that heavy metals and environmental microbiota act synergistically to modulate the gut microbiome structure of exposed children, with environmental microbiota serving as a critical mediator in the combined effects of heavy metals and environmental microbes on the pediatric gut microbiome 114. Thus, enhanced environmental protection and reduced exposure to various pollutants are essential for the effective prevention of COPD.

### Aging

Aging drives COPD-associated gut microbiota dysbiosis through multi-dimensional mechanisms, which can be clearly divided into two categories: changes induced by aging itself, and those that directly interact with COPD pathogenesis. First, aging itself alters the gut microbiota through physiological, immunological, and pharmacological pathways. At the physiological level, reduced gastric acid secretion in the elderly leads to significant differences in acid-tolerant bacteria such as *Bifidobacterium* between young and elderly populations 115, 116. Meanwhile, slowed intestinal peristalsis promotes the overproliferation of opportunistic pathogens including *Escherichia coli*, disrupting the native balance of the gut microbiota 117. At the immune level, aging impairs intestinal mucosal immune function, reducing the ability to recognize and clear harmful bacteria 118. Elevated pro-inflammatory cytokines further create an inflammatory microenvironment that decreases the abundance of inflammation-sensitive beneficial bacterial groups such as *Bacteroides fragilis* 119. In addition, long-term antibiotic use in elderly individuals causes non-selective suppression of beneficial bacteria including *Lactobacillus* 120. Furthermore, these aging-related gut microbial changes directly interact with COPD pathological processes. They overlap with the progression of COPD, exacerbate intestinal microecological disorders via the gut-lung axis, and ultimately exert adverse effects on the development and progression of COPD 121.

### Obesity

Studies have found that obesity not only reduces an individual’s quality of life and impairs bodily function but also is associated with an increased risk of developing COPD 122. A growing body of research has shown that gut microbiota dysbiosis frequently occurs in obese individuals 123. Backhed *et al*. proposed that the gut microbiota may regulate energy storage through host signaling pathways. Despite reduced food intake, adult germ-free mice still exhibit a 57% increase in total body fat content compared to conventionally raised mice, which suggests that gut microbes play a crucial role in regulating energy metabolism as well as fat synthesis and conversion 124. G protein-coupled receptor 41 (GPR41) is a SCFA receptor 125. Mice deficient in GPR41 exhibit reduced levels of the intestinal hormone peptide YY (PYY), slowed intestinal transit function, and decreased SCFA absorption. The PYY hormone plays a role in appetite control, and reduced levels of this substance may lead to an increased risk of obesity 126, 127. This “obesity - gut microbiota metabolic dysregulation - decreased SCFAs” phenomenon interacts with the chronic inflammation of COPD and drives disease progression through the gut-lung axis. Therefore, we hypothesize that gut microbiota dysbiosis induced by obesity is associated, to a certain extent, with the development and progression of COPD.

## MECHANISMS OF THE GUT MICROBIOTA IN THE ONSET AND PROGRESSION OF COPD

The gut microbiota is not an isolated microbial community; instead, it forms a close functional connection with the respiratory system through the “gut-lung axis”. Imbalances in the structure of the gut microbiota and abnormalities in its metabolites in patients with COPD can affect the pathophysiological processes of the lungs through multiple pathways such as immune regulation, inflammatory responses, and barrier function, thereby emerging as a key driving factor in the onset and progression of the disease.

### The gut microbiota reduces inflammation and participates in immune responses in COPD

A large body of research has confirmed that the gut microbiota can reduce inflammation induced by COPD through multiple pathways and targets. It is well known that cigarette smoke - exposed mice serve as a classic animal model for COPD research, and the gut commensal *Parabacteroides goldsteinii* (Pg) strain MTS01 can reduce pulmonary and intestinal inflammation in CS-exposed mice. Specifically, Pg MTS01 not only significantly upregulates the antioxidant activity of cells but also enhances the activity of ribosomes and mitochondria within cells 128. A study by Wang *et al*. found that Xuanbai Chengqi Decoction alleviates the exacerbation of COPD by enriching gut microbiota such as *Gordonibacter*, *Allobaculum*, and *Akkermansia spp*. This process corrects the Th17/Treg imbalance, reduces the expression of TNF- α , IL-1 β , and matrix metalloproteinase-9 (MMP-9), and inhibits the infiltration of inflammatory cells 129. In addition, SCFAs produced by the gut microbiota can inhibit the activation of the NF- κ B pathway in alveolar macrophages through the PPAR γ pathway, enhance the production of regulatory T cells, and suppress the differentiation of Th17 cells 130, thereby alleviating the symptoms of IBD. However, whether they can alleviate IBD induced by COPD warrants further investigation. Similarly, butyrate, a member of the SCFA family, can also reduce inflammatory responses in patients with COPD by inhibiting histone deacetylase (HDAC) activity and regulating Treg production 131–133. Notably, as a core molecule mediating chronic inflammation in COPD, the (NOD-like receptor protein 3) NLRP3 inflammasome plays a pivotal hub role in the regulation of COPD inflammatory responses by the gut microbiota. The NLRP3 inflammasome is a key pro-inflammatory activation receptor in the innate immune system, and it exhibits abnormally excessive activation in patients with COPD 134. Moreover, the mRNA levels of NLRP3, caspase-1, IL-18 and IL-1 β are elevated in patients with AECOPD compared with smokers, while they are decreased in patients with stable COPD, indicating an association between the NLRP3 inflammasome and AECOPD 135. Mao *et al.* found that Bufei Jianpi formula (BJF) upregulated the expression of GPR43 and downregulated the expressions of NLRP3, caspase-1, IL-1 β and IL-18 in COPD rats. Furthermore, BJF could enhance the intestinal mucosal immune function by regulating the intestinal SCFAs/GPR43/NLRP3 pathway, thereby ameliorating the alterations of the intestinal microbiome and intestinal metabolome 136. In summary, the NLRP3 inflammasome serves as a core hub for the regulation of COPD inflammation by the gut microbiota, and the gut-lung axis mediated microbiota-inflammasome regulatory network plays a critical role in the progression of COPD, thereby providing important theoretical support for the clinical targeted intervention of COPD.

Eosinophils have been identified as a biomarker for COPD 137. In previous studies, it has been found that the gut microbiome is crucial for shaping the host’s immune system 138, 139. These microorganisms are not involved in the progression of COPD through pathogenic infection; instead, they indirectly affect the function of the host’s immune system by regulating the number of eosinophils in human blood 63. Study results have confirmed that an increase in the number of eosinophils in the blood is positively correlated with the exacerbation of COPD, a decrease in FEV values, and an increase in mortality 140. The abundance of *Bacteroidetes* in the intestine is not only associated with the proportion of eosinophils in the blood of COPD patients but also can reflect the patients’ lung function status to a certain extent 63.

### The gut microbiota can alleviate emphysema and airway remodeling caused by COPD

The gut microbiota primarily alleviates COPD - associated emphysema (characterized by alveolar structural destruction and decreased lung tissue elasticity) and airway remodeling (characterized by airway smooth muscle hyperplasia, collagen deposition, and mucous gland hypertrophy) through multiple dimensions of the “gut-lung axis” 141. In terms of metabolite regulation, SCFAs, as key molecules, bind to GPR41/43 receptors on lung tissue cells to activate the AMPK pathway on the one hand, and inhibit the phosphorylation of NF- κ B and MAPK to reduce the release of pro-inflammatory factors such as TNF- α and IL-6 on the other, thereby alleviating inflammatory damage to the lungs and inflammation-driven tissue hyperplasia 142, 143. Specifically, acetate, the primary component of SCFAs derived from the gut microbiota, can bind specifically to the GPR43 receptor on the surface of airway epithelial cells and activate the downstream AMPK signaling pathway, thereby effectively maintaining the structural integrity of airway epithelial tight junctions, inhibiting the release of chronic pulmonary inflammatory factors and the infiltration of inflammatory cells, and alleviating airway epithelial injury 144. In terms of immune balance regulation, beneficial gut microbiota (such as *Bifidobacterium* and *Lactobacillus*) induce the differentiation of naive T cells into Treg via intestinal dendritic cells (DCs) 145–147. After migrating to lung tissue, Treg secrete transforming growth factor- β (TGF- β ) and IL-10 148, which inhibit the pro-inflammatory response mediated by Th17 cells 149. In terms of oxidative stress inhibition and mitochondrial protection, the gut microbiota metabolically produces glutathione (GSH) precursors and activates the pulmonary Nrf2 pathway, upregulating antioxidant enzymes such as superoxide dismutase (SOD) and glutathione peroxidase (GSH-Px) to scavenge reactive oxygen species (ROS) 150–152. Its metabolites (*e.g.*, acetate) can also clear damaged mitochondria by regulating mitophagy, while SCFAs activate the AMPK pathway to promote mitochondrial biogenesis 153. These processes protect the functions of alveolar epithelial cells and airway smooth muscle cells, alleviate oxidative damage to lung tissue, and ultimately, the gut microbiota corrects dysbiosis through the aforementioned mechanisms, breaks the “intestinal dysregulation - pulmonary damage” vicious cycle, and achieves the effect of alleviating related pathological changes.

### The gut microbiota can enhance the intestinal epithelial barrier function impaired by COPD

*Lactobacilli*, *Bifidobacteria*, *E. coli*, and next-generation probiotics exhibit effects of maintaining intestinal epithelial homeostasis and promoting health by regulating intestinal epithelial cells (IECs) through their surface compounds 154. Surface components of intestinal probiotics, such as flagella, pili, surface layer proteins, capsular polysaccharides, lipoteichoic acid, and LPS, constitute microbe-associated molecular patterns (MAMPs) 155. They can specifically bind to pattern recognition receptors (PRRs), such as nucleotide-binding oligomerization domain - like receptors (NLRs) and Toll-like receptors (TLRs) 156, and regulate NF- κ B, MAPK, PPAR γ , and other signaling pathways in IECs 157. In addition, MAMPs also regulate cell protease-dependent signaling cascades and induce the production of various cytokines and chemokines to alleviate inflammation and enhance intestinal epithelial function 158.

Some metabolites produced by the gut microbiota, such as secreted proteins, organic acids, indoles, bacteriocins, hydrogen peroxide (H ${}_{2}$ O ${}_{2}$ ), and nitric oxide (NO), protect the intestinal epithelial barrier by promoting mucus secretion from goblet cells, increasing the production of antimicrobial peptides, or enhancing the expression of tight junction proteins 159. SCFAs play a crucial role in intestinal mucosal immunity. They enhance the metabolism of plasma B cells, promote the differentiation of goblet cells and the production of mucins, and increase the synthesis of immunoglobulin A in the intestine, thereby further strengthening the intestinal epithelial barrier function 160. In particular, butyrate can upregulate the expression of tight junction proteins including claudin-1 161, claudin-3, claudin-4 69, occludin, and ZO-1, while downregulating the expression of claudin-2 162 to restore the intestinal epithelial barrier function 163, 164.

## PREVENTION AND TREATMENT STRATEGIES FOR COPD BASED ON GUT MICROBIOTA REGULATION

**Figure 3:**
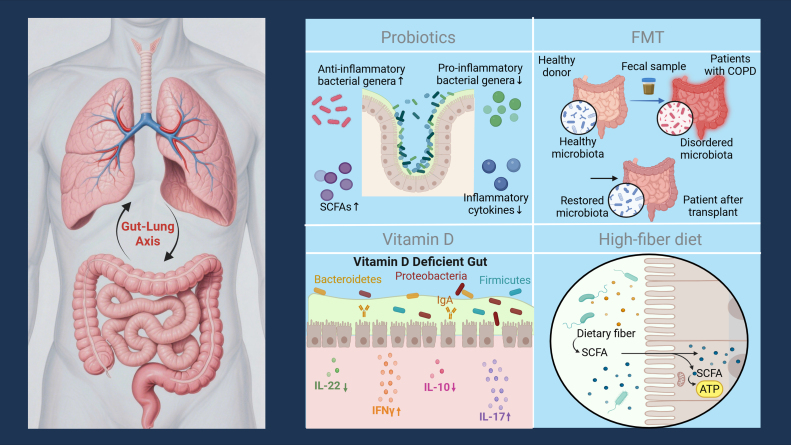
COPD Prevention and Treatment Strategies Based on Gut Microbiota. This schematic diagram shows how probiotics, fecal microbiota transplantation (FMT), vitamin D, and a high-fiber diet regulate the intestinal microbiota via the gut-lung axis to intervene in or prevent COPD.

With the in-depth research on the “gut-lung axis” theory, the gut microbiota has become a potential new target for the prevention and treatment of COPD. By regulating the structure of the gut microbiota and restoring the balance of gut microbial metabolism, the dysfunction of the gut-lung axis can be improved at the source, providing new insights for the prevention, treatment, and management of acute exacerbations of COPD. Currently, gut microbiota-based prevention and treatment strategies mainly include probiotic intervention, dietary regulation, fecal microbiota transplantation, and targeted metabolite supplementation, and their clinical application value is gradually being verified (Figure 3).

### Probiotics

As live microorganisms beneficial to the host’s health, probiotics can improve the structure of the gut microbiota by supplementing missing anti-inflammatory bacterial genera and inhibiting the excessive proliferation of pro-inflammatory bacteria, making them the most widely studied gut microbiota regulation approach currently 165. Multiple clinical studies have confirmed that supplementing probiotics such as *Bifidobacterium* and *Lactobacillus* in patients with COPD can significantly increase the diversity of the gut microbiota and reduce the abundance of pro-inflammatory bacteria such as *E. coli*^166^. A randomized controlled trial showed that after stable COPD patients supplemented with probiotics daily, the level of SCFAs in their feces increased, the serum levels of IL-6 and TNF- α decreased, and the percentage of FEV ${}_{1}$ % pred was higher than that in the control group 167. The intervention effect of probiotics is closely related to strain selection, dosage, and intervention timing. Combined probiotics (*e.g.*, *Bifidobacterium* + *Lactobacillus* + *Enterococcus faecalis*) can improve the gut microbiota structure more comprehensively than a single strain. Additionally, adjuvant probiotic therapy during the acute exacerbation phase can reduce gut microbiota dysbiosis caused by antibiotic use in patients and lower the risk of recurrent disease exacerbation 168. Notably, the therapeutic effects of probiotics are characterized by obvious strain specificity, and significant variability in response exists among different individuals. In the future, it will be necessary to combine gut microbiota detection results to achieve precise intervention.

### Fecal microbiota transplantation (FMT) and precision targeting

As a potent approach for reconstructing the gut microbiota, FMT can transfer the complete gut microbiota structure from healthy donors to the intestines of patients, thereby achieving the restoration of the overall functional capacity of the gut microbiota 169. It is important to acknowledge that, however, the evidence base supporting FMT application in COPD remains limited and is largely derived from preclinical animal studies and early-phase clinical investigations. Animal studies have shown that after transplanting the fecal microbiota from healthy mice to COPD model mice, the degree of pulmonary inflammation in the model mice was significantly reduced, the destruction of alveolar structure was improved, and the intestinal barrier function and immune status were simultaneously restored 170. Global multiple clinical trials on FMT for COPD patients are currently underway, but all remain in the volunteer recruitment phase; however, its long-term safety and donor microbiota matching remain key issues that require focused attention 171.

With the advancement of metagenomics and metabolomics technologies, precision intervention strategies targeting specific gut microbiota or metabolic pathways have gradually emerged. These targeted precision approaches represent key areas of ongoing and future research, with their clinical translation and practical application for COPD still under active investigation—framing important directions for subsequent translational and clinical studies. For instance, approaches such as the targeted elimination of overproliferating pro-inflammatory bacterial genera (*e.g.*, *E. coli*) using bacteriophages, or the supplementation of recombinant gut microbial metabolic enzymes to promote SCFA synthesis, exhibit higher specificity and controllability 172. For patients with abnormal tryptophan metabolic pathways, supplementation with precursors of gut microbial metabolites such as indole derivatives can alleviate pulmonary inflammation by activating AhR signaling pathway, providing a new direction for the precise prevention and treatment of COPD 173.

### Vitamin D

Vitamin D is involved in multiple physiological processes by regulating approximately 3% of the human genome, and its immunomodulatory function is closely associated with the pathological mechanisms of COPD 174, 175. At the molecular level, Vitamin D can directly interact with the NF- κ B and p38/MAPK pathways. By inhibiting the activity of pro-inflammatory transcription factors, it reduces the transcriptional release of cytokines such as IL-6 and TNF- α , as well as chemokines, thereby blocking the inflammatory cascade 176. This anti-inflammatory effect holds significant importance in COPD driven by chronic inflammation. A bidirectional regulatory “metabolic crosstalk” exists between Vitamin D and the gut microbiota: On one hand, sufficient Vitamin D can significantly enhance the diversity of the gut microbiota. For example, experiments on C57BL/6 mice showed that the diversity of colonic microbiota in the Vitamin D-sufficient group was 50 times that of the Vitamin D-deficient group 177; it can also promote the proliferation of anti-inflammatory bacterial genera and improve gut microbiota homeostasis 178. On the other hand, metabolites of the gut microbiota can affect the activation and absorption of Vitamin D. Gut microbiota dysbiosis may exacerbate Vitamin D deficiency (VDD), with the prevalence of VDD in the population as high as 80%. Furthermore, VDD and chronic inflammation exhibit a bidirectional causal relationship 179. In COPD, VDD is closely associated with disease progression. Clinical studies have confirmed that after supplementing high-dose Vitamin D to COPD patients with severe VDD, the frequency of acute exacerbations is significantly reduced 180. The level of serum 25-hydroxyvitamin D (25-OHD, a core biomarker for Vitamin D exposure) is significantly negatively correlated with inflammatory markers such as CRP and IL-6, suggesting that it may improve the inflammatory state of COPD through the dual pathways of anti-inflammation and gut microbiota regulation 181.

### High-dietary-fiber diet

A high-dietary-fiber diet exerts positive effects through the “diet-microbiota-immunity” axis. Its intake is negatively correlated with the activity of CRP, an acute inflammatory marker, and can reduce systemic inflammation and the predisposition to COPD. The core mechanism lies in promoting the gut microbiota to produce SCFAs with anti-inflammatory properties 182. As a key energy source for the gut microbiota, dietary fiber can significantly alter the microbiota structure, reduce the *Firmicutes*/*Bacteroidetes* (F/B) ratio, and increase local SCFA concentrations 183. Clinical and epidemiological studies have confirmed that reduced lung function is associated with insufficient dietary fiber intake, and appropriate intake can lower the incidence of COPD in smokers 184. A female cohort study in Sweden showed that long-term high-fiber diet was associated with a 30% reduction in COPD risk 185. Animal experiments further verified that a high-fiber diet containing non-fermentable cellulose and fermentable pectin can alleviate the progression of emphysema and inflammatory responses induced by cigarette smoke in mice, while simultaneously increasing gut microbiota diversity and metabolic activity 186.

## CONCLUSION

As a complex, progressive respiratory disease, the exploration of the pathological mechanisms of COPD has expanded from traditional local lung research to the field of systemic microecological regulation. Based on the theoretical framework of the gut-lung axis, this article systematically reviews the association between the gut microbiota and COPD as well as the research progress, with the core conclusions summarized as follows: First, the gut-lung axis establishes a close pathological connection between the intestine and the lung through multiple pathways such as immune regulation and metabolic crosstalk, serving as a key perspective for interpreting the systemic pathological characteristics of COPD. Second, COPD patients generally exhibit gut microbiota dysbiosis, characterized by a reduction in probiotics, enrichment of opportunistic pathogens, and a decrease in microbiota diversity, accompanied by abnormal changes in characteristic metabolites such as SCFAs and tryptophan metabolites. Third, factors such as smoking, malnutrition, and aging disrupt the homeostasis of the intestinal microecology, driving microbiota dysbiosis to participate in the occurrence and development of COPD. Fourth, the gut microbiota plays an important role in the progression and acute exacerbation of COPD by regulating systemic chronic inflammation, immune balance, and restoring the function of the intestinal epithelial barrier. Collectively, these findings reveal the core value of the gut microbiota as a new target in the research on the pathological mechanisms of COPD.

Although phase-specific progress has been achieved in research on the association between the gut microbiota and COPD, further in-depth exploration across multiple dimensions is still needed in the future. Regarding the molecular regulatory mechanisms of the gut-lung axis, research can focus on the specific binding mechanisms between gut microbial metabolites and surface receptors of lung cells. By simulating the intestinal-lung signal transmission process using organoid co-culture models, it is necessary to via integrated analysis of gut and lung microbiota clarify how microbial metabolites regulate chronic pulmonary inflammation through activating signaling pathways such as NF- κ B and NLRP3 inflammasome, and to reveal the precise regulatory network of the “microbiota – metabolite - host receptor” axis in the progression of COPD.

In the research on specific gut microbiota biomarkers, it is necessary to rely on multi-center, large-sample COPD cohorts, combine metagenomic sequencing with machine learning algorithms, and screen gut microbiota characteristic profiles that can accurately distinguish different disease stages of COPD, risk stratification of acute exacerbations, and differences in treatment responses. Emphasis should be placed on verifying the dynamic association between changes in the abundance of specific bacterial genera and lung function indicators, thereby promoting the use of microbiota biomarkers as auxiliary tools for the precise diagnosis and treatment of COPD. In response to the heterogeneity of gut microbiota in COPD patients, personalized intervention strategies based on microbiota detection results should be explored. For example, for patients deficient in SCFA - producing bacteria, develop probiotic preparations targeting the supplementation of acid-producing bacterial strains; for individuals with impaired intestinal barrier function, combine prebiotics with mucosal repair agents to enhance intervention efficacy. Meanwhile, long-term follow-up studies should be conducted to assess the efficacy of microbiota-based interventions in reducing the frequency of COPD acute exacerbations and improving quality of life scores, with priority given to extended human studies that concurrently characterize both gut and lung microbiota to validate their translational potential in clinical practice.

Innovation in the application of multi - omics integration technology is also crucial. It is necessary to establish a multi - omics database encompassing “gut microbiota metagenome - blood metabolome - lung transcriptome”, identify key association nodes among the three through bioinformatics analysis, and use single-cell spatial transcriptomics to localize specific gene expression regions in pulmonary inflammatory cells that are regulated by microbial metabolites. This will provide a novel perspective for deciphering the cellular and molecular basis of cross - organ regulation in the gut - lung axis.

Notably, we should also focus on differential research on the gut microbiota of special populations: analyzing the gut microbiota characteristics of elderly COPD patients and COPD patients with comorbid diabetes/cardiovascular diseases, exploring the impact of age and comorbidity status on gut - lung axis crosstalk, while investigating the dynamic restoration patterns of the gut microbiota in long-term smoking COPD patients after smoking cessation, and evaluating the synergistic effect of smoking cessation combined with microbiota intervention on lung function recovery. This will provide a basis for formulating targeted prevention and treatment strategies for special populations. In summary, through the in - depth integration of basic research and clinical practice in the future, we are expected to translate gut microbiota regulation into a novel strategy for COPD prevention and treatment, offering a new breakthrough point for improving patients’ quality of life and delaying disease progression.

## AUTHORS CONTRIBUTIONS

**Simin Yang:** Writing – original draft, **Shuting Zeng:** Writing – review and editing, **Yongan Deng:** Writing – original draft, **Xiaodong Duan:** Formal analysis, **Chengkai Chen:** Conceptualization, **Luyun Sun:** Formal analysis, **Yongkang Qiao:** Writing – original draft, **Zunpeng Shu:** Conceptualization, Writing – review and editing, Supervision, Project administration, Funding acquisition.

## CONFLICT OF INTEREST

The authors declare no conflict of interest.

## ABBREVIATIONS

AECOPD – acute exacerbation COPD

AhR – aryl hydrocarbon receptor

BA – bile acid

BJF – Bufei Jianpi formula

COPD – Chronic Obstructive Pulmonary Disease

CS – cigarette smoke

DMB – 3,3-dimethyl-1-butanol

FMO3 – flavin-containing monooxygenase 3

FMT – fecal microbiota transplantation

GERD – gastroesophageal reflux disease

HPA – Hypothalamic-Pituitary-Adrenal

IBD – inflammatory bowel disease

MAMPs – microbe-associated molecular patterns

PASMC – pulmonary artery smooth muscle cell

PH – pulmonary hypertension

TMA – trimethylamine

TMAO – Trimethylamine N-oxide

VDD – Vitamin D deficiency
